# Evaluation of the predictive values of collapse and necrotic lesion boundary for osteonecrosis of the femoral head prognosis

**DOI:** 10.3389/fendo.2023.1137786

**Published:** 2023-03-13

**Authors:** Yinuo Fan, Xuejie Liu, Yuan Zhong, Jiahao Zhang, Yuhao Liu, Hanjun Fang, Wei He, Chi Zhou, Zhenqiu Chen

**Affiliations:** ^1^ The Third Clinical of Medical School, Guangzhou University of Chinese Medicine, Guangzhou, Guangdong, China; ^2^ School of Pharmaceutical Sciences, Guangzhou University of Chinese Medicine, Guangzhou, Guangdong, China; ^3^ The First Clinical of Medical School, Guangzhou University of Chinese Medicine, Guangzhou, Guangdong, China; ^4^ The Department of Orthopedics, The First Affiliated Hospital of Guangzhou University of Chinese Medicine, Guangzhou, Guangdong, China; ^5^ The Department of Orthopedics, The Third Affiliated Hospital of Guangzhou University of Chinese Medicine, Guangzhou, Guangdong, China

**Keywords:** osteonecrosis of the femoral head, necrosis lesion boundary, prognosis, collapse, the necrosis lesion boundary classification

## Abstract

**Objective:**

Osteonecrosis of the femoral head (ONFH) is a disabling and intractable orthopedic disease largely affecting young and middle-aged groups. Current standard of treatment relies on the collapse of femoral head as a predictor for prognosis. However, a wide range of variability in repair potentials is observed in patients with femoral head collapse. Therefore, the present study aimed to evaluate the accuracy of femoral head collapse as a predictor and to propose the necrotic lesion boundary as a novel yet reliable measure for ONFH prognosis.

**Methods:**

A retrospective cross-sectional study was conducted at the First Affiliated Hospital of Guangzhou University of Chinese Medicine, 203 hips with ONFH from 134 patients were included. The occurrences and progression of femoral head collapse were recorded. Necrosis lesion boundary was quantified and classified for each case based on anteroposterior view intact ratio (APIR) and the frog-leg view intact ratio (FLIR) as independent variables. Dependent variables were defined as progressive collapse or terminal collapse for Association Research Circulation Osseous (ARCO) stage II and III respectively. Logistic regression analysis, Receiver Operating Characteristic (ROC) curve and Kaplan-Meier (K-M) survival analysis was performed and results were interpreted.

**Results:**

Out of the 106 hips in ARCO stage II, 31 hips collapsed with further progression, while 75 hips had no collapse or collapse with repair of the necrotic areas. Out of the 97 hips in ARCO stage IIIA, the collapse continued to progress in 58 hips while the necrotic areas were repaired in 39 hips. Logistic regression analysis demonstrated that both APIR and FLIR, were independent risk factors. Further ROC curve analysis indicated that the cutoff values of APIR and FLIR could be considered as indications for evaluating the prognosis of ONFH. Contrary to the traditional view of poor prognosis after femoral head collapse, K-M survival analysis demonstrated a high value of APIR and FLIR for ONFH prognosis.

**Conclusion:**

The present study found that the occurrence of collapse is an oversimplified predictor for ONFH prognosis. The collapse of the femoral head in ONFH does not predict a poor prognosis. The necrosis lesion boundary has a high value in predicting ONFH prognosis and informing clinical treatment strategies.

## Introduction

Osteonecrosis of the femoral head (ONFH) is a disabling and intractable orthopedic disease largely affecting young and middle-aged groups (1). ONFH is characterized by the collapse of the articular surface of the femoral head due to insufficient bone remodeling capacity and disruption of the vascular supply (2). Middle and late stages of ONFH can further lead to the collapse of femoral head’s articular surface. As a result, recent studies have argued that the severity of femoral head collapse should be the ultimate guidance to clinical treatments for ONFH and have found reliable methods to assess or predict femoral head collapse (3–12).

In current clinical practice, femoral head collapse is a marker of severe irreversible osteonecrosis and is an indication for hip preservation surgery or total hip arthroplasty (THA) (13–21). However, we found contradictory conclusions in our clinical observations of patients with ONFH. While some developed further collapse as predicted, cases in Association Research Circulation Osseous (ARCO) stage II were observed to undergo repair of the necrotic area which eventually led to regained abilities for activities of daily life in patients. On the contrary, some patients developed further collapse and underwent THA in as short as 1 year. More interestingly, in the follow-up of the patients of ARCO stage IIIA, we found a situation similar to the ARCO stage II described above. Moreover, a study conducted by He et al. which employed a non-surgical approach for patients in ARCO stage II, with a mean follow-up of 7.95 years, found that progression to ARCO stage III were either halted (in 1/4 of the patients), or prevented with timely non-surgical interventions (22). These findings contradict the traditional view on poor prognosis after femoral head collapse and provide possibilities for non-surgical interventions.

Since we observed a high correlation between the anterolateral necrotic boundary and the collapse of femoral head (12), we hypothesized that the necrotic lesion boundary, as a finer feature of the necrosis, better predicts the prognosis of ONFH. To investigate this hypothesis, we conducted a retrospective cross-sectional study to evaluate the predictive value of collapse and necrotic lesion boundary for ONFH prognosis.

## Methods

### Patients

This study was approved by the institutional review board. Patients with ONFH who presented to the First Affiliated Hospital of Guangzhou University of Chinese Medicine from January 2000 to December 2016 were included. The diagnosis of ONFH was confirmed by senior doctors from X-ray and MRI based on the Chinese Guideline for the Diagnosis and Treatment of Osteonecrosis of the Femoral Head in Adults (23).

### Inclusion criteria

Inclusion criteria were as follows: (i) patients who presented with ARCO stage II or IIIA (based on ARCO classification standard established in 2019); (ii) patients aged between 18 years and 55 years; (iii) patients with regular follow-up and complete imaging data of hip X-rays taken at least once every 6 months. Both anteroposterior (AP) view and frog-leg lateral (FL) view of both hips are required.

### Exclusion criteria

Exclusion criteria were as follows: (i) patients who underwent hip preservation surgery during the follow-up; (ii) patients with severe cardiovascular and cerebrovascular diseases, tumors, infections, or mental health deficit; (iii) patients who had coxa plana, congenital hip dysplasia or other diseases that affect the normal physiological structure of the hip joint; (iv) patients who had rheumatoid arthritis, ankylosing spondylitis and other rheumatic diseases involving the hip joint; and (v) patients who continued to take glucocorticoids or drink alcohol.

### Imaging protocols and measurement of predictive indicators

**Figure 1:**
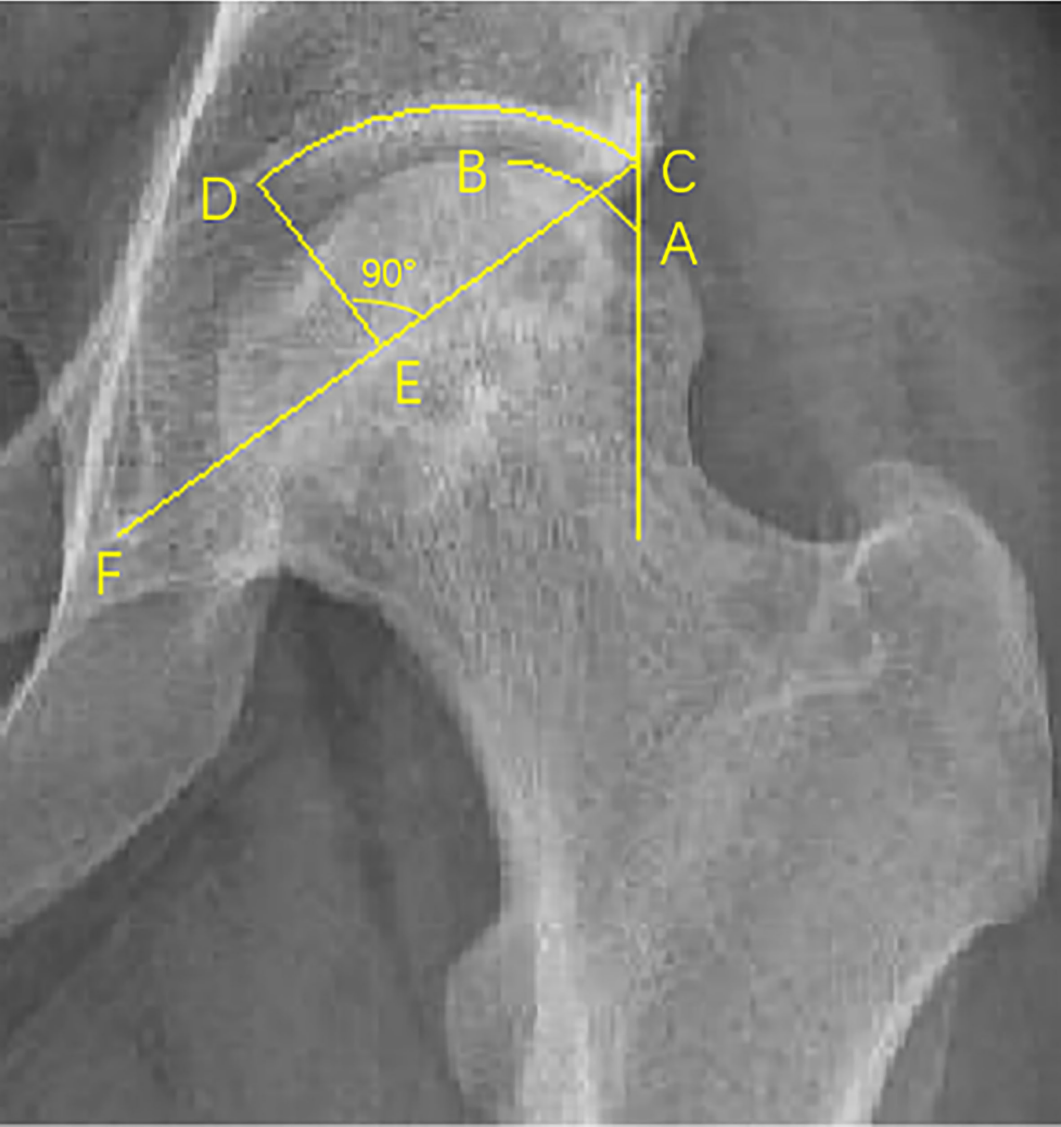
The measurement of IR. Point C is the outermost edge of the acetabulum, point F is the lowest point of the teardrop, and point D is the intersection of the vertical line of the midpoint of the CF line and the acetabulum. The parallel line with the central axis through point C intersects the femoral head at point A, and B is the outer boundary of the necrotic area involving the wall of the femoral head. Length (A-B) represents the contour length of the femoral head in the non-necrotic area of the weight-bearing part. Length (CD) represents the contour length of the acetabulum in the weight-bearing part. APIR/FLIR= Length (A-B)/Length (CD)×100%. Length (C-D) as an acetabular weight-bearing part does not change, while Length (A-B) changes according to the location of the necrotic lesion boundary. Smaller lengths (A-B) imply smaller APIR and FLIR, while smaller APIR and FLIR values indicate that the necrotic lesion boundary is closer to the outer edge of the acetabulum, which means less intact femoral head is available to bear weight.

The anteroposterior view intact ratio (APIR) and the frog-leg view intact ratio (FLIR) were used to assess the retention of the anterolateral boundary of the necrotic lesion. The necrosis lesion boundary was quantified by APIR and FLIR (**Figure 1**). To establish reliability, APIR and FLIR were blindly measured by two independent observers. Additionally, APIR and FLIR were unified into the combined intact ratio (CIR) by means of logistic regression analysis.

### Classification of the necrotic lesion boundary

**Figure 2:**
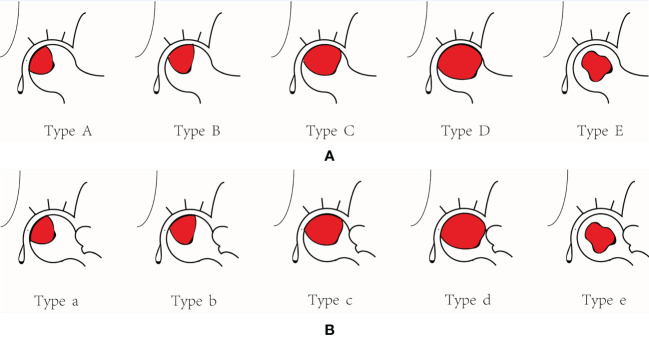
The necrosis lesion boundary classification. **(A) **The lateral necrosis lesion boundary classification. **(B) **The anterior necrosis lesion boundary classification.

Our study reclassified ONFH by quantitative values of the necrosis lesion boundary. According to the proportion of the non-necrotic area of the weight-bearing part of the femoral head in the weight-bearing area of the acetabulum corresponding to the AP view, the lateral necrotic boundary is classified (**Figure 2A**). (i) type A: Point B (the boundary point of the necrotic area on the femoral head) is inside the weight-bearing area, that is, the non-necrotic area in the weight-bearing part of the femoral head occupies more than 2/3 of the weight-bearing area of the acetabulum (arc AB/arc CD=APIR>2/3); (ii) type B: the non-necrotic area in the weight-bearing part of the femoral head occupies between 1/3-2/3 of the weight-bearing area of the acetabulum (1/3<APIR<2/3); (iii) type C: the non-necrotic area in the weight-bearing part of the femoral head occupies no more than 1/3 of the weight-bearing area of the acetabulum (APIR<1/3); (iv) type D: the necrosis lesion boundary extends beyond the outer rim of the acetabulum; and (v) type E: the necrotic lesion boundary is inside the femoral head. According to the proportion of the non-necrotic area of the weight-bearing part of the femoral head in the weight-bearing area of the acetabulum corresponding to the FL view, the anterior necrotic boundary is classified (**Figure 2B**). (i) type a: the non-necrotic area in the weight-bearing part of the femoral head occupies more than 2/3 of the weight-bearing area of the acetabulum (arc AB/arc CD=FLIR>2/3); (ii) type b: the non-necrotic area in the weight-bearing part of the femoral head occupies between 1/3-2/3 of the weight-bearing area of the acetabulum (1/3<FLIR<2/3); (iii) type c: the non-necrotic area in the weight-bearing part of the femoral head occupies no more than 1/3 of the weight-bearing area of the acetabulum (FLIR<1/3); (iv) type d: the necrosis lesion boundary extends beyond the outer rim of the acetabulum; and (v) type e: the necrosis lesion boundary is inside the femoral head.

### Classification of outcomes

(i) For ARCO stage II, progressive collapse as an endpoint was defined as persistent collapse without repair (collapse >4 mm or progression to ARCO stage IV) at follow-ups; and(ii) For ARCO stage IIIA, terminal collapse as an endpoint was defined as persistent collapse without repair (collapse >4 mm or progression to ARCO stage IV) on X-rays. The degree of collapse was measured according to Nishii’s modified method (24).

### Grouping of patients

According to the severity of femoral head collapses, patients in ARCO stage II were separated into a progressive collapse group and non-progressive collapse group (non-progressive collapse group included patients with no collapse). Patients in ARCO stage IIIA were separated into a terminal collapse group and a necrotic repair group based on the repair progress of the femoral head.

### Statistical analysis

All analyses of data were conducted with SPSS Software version 23.0 (International Business Machines, Armonk, New York, USA) and GraphPad Prism Software version 7.04 (GraphPad Software Inc, San Diego, CA, USA). Measurement data were tested for normality using the Kolmogorov-Smirnov method. Measurement data subject to normal distribution were expressed as mean ± standard deviation (Mean ± SD), and independent samples t-test was performed, and variables that did not obey normal distribution were expressed as median and interquartile range [M (P25], P75)]. The independent samples t test was used to compare the measurement data that obeyed the normal distribution between groups, and the nonparametric two independent sample Mann-Whitney U test was used for the non-normal distribution. The count data were expressed as frequency and analyzed by chi-square test. Univariate and multivariate logistic regression analysis were performed to identify the risk coefficient of the variable, and then the receiver operating characteristic (ROC) curve was used to evaluate the cutoff of the variable. The Kaplan-Meier (K-M) survival analysis was performed with the classification of outcomes. All statistical analyses were two-sided, with p-values < 0.05 indicating statistical significance.

## Results

A total of 203 hips from 134 patients were included in this study, including 106 hips in ARCO stage II and 97 hips in ARCO stage IIIA. According to the necrosis lesion boundary classification, there were 3 type A hips, 21 type B hips, 133 type C hips, 26 type D hips, 20 type E hips, 1 type a hip, 28 type b hips, 128 type c hips, 28 type d hips and 18 type e hips. 113 hips were diagnosed with steroid-induced ONFH (SONFH), 70 hips with alcohol-associated ONFH (AONFH), and 20 hips with idiopathic ONFH (IONFH).

Out of the 106 hips in ARCO stage II, 31 hips collapsed with further progression while 75 hips had no collapse or collapse with repair of the necrotic areas. Out of the 97 hips in ARCO stage IIIA, the collapse continued to progress in 58 hips while the necrotic areas were gradually repaired in 39 hips. For both ARCO stage II and IIIA, the necrotic lesion boundary classification turned out to be a key factor affecting the outcome in both the progressive collapse and terminal collapse groups (P=0.000, **Table 1**). Non-parametric tests further showed that there were significant statistical differences in both APIR and FLIR between the progressive collapse group and the non-progressive collapse group, and between the terminal collapse group and the necrotic repair group (P=0.000, **Tables 2**, **3**). For the progressive collapse or terminal collapse groups, logistic regression analyses showed that both APIR (progressive collapse: OR 0.886, 95% CI 0.801-0.980, p=0.018/terminal collapse: OR 0.823, 95% CI 0.733-0.924, p=0.001) and FLIR (progressive collapse: OR 0.783, 95% CI 0.690-0.888, p=0.000/terminal collapse: OR 0.941, 95% CI 0.893-0.992, p=0.025) were independent risk factors, and they inversely predicted the risk of collapse (**Table 4**).

**Table 1:** The correlation between the characteristics of ARCO stage II-IIIA and collapse.

	Progressive collapse group (31 hips)	Non-progressive collapse group (75 hips)	*P* value	Terminal collapse group (58 hips)	Necrotic repair group (39 hips)	*P* value
Age			0.554			0.385
18-35	11	35		25	19	
36-55	17	33		25	18	
>55	3	7		8	2	
Sex			0.561			0.188
Males	21	55		43	24	
Females	10	20		15	15	
Associated factor			0.603			0.181
Corticosteroid	19	38		29	27	
Alcohol	9	27		24	10	
Idiopathic	3	10		5	2	
The lateral necrosis lesion boundary classification			0.000			0.000
A	0	3		0	0	
B	2	10		1	7	
C	23	45		35	30	
D	6	0		22	1	
E	0	17		0	1	
The anterior necrosis lesion boundary classification			0.000			0.000
A	0	1		0	0	
B	2	18		3	6	
C	24	39		35	30	
D	5	0		20	0	
E	0	17		0	3	
Follow-up time (mths)	37 (5-94)	84 (60-241)	/	21 (6-78)	80 (60-154)	/

**Table 2:** The correlation between APIR and collapse.

	Hips	APIR (%)	z	*p*
Progressive collapse				
Progressive collapse group	25	15.18 (7.72,27.13)	4.407	0.000
Non-progressive collapse group	56	29.06 (23.97,32.66)		
Terminal collapse				
Terminal collapse group	34	12.31 (8.26,15.54)	-5.977	0.000
Necrotic repair group	35	23.31 (18.84,31.29)		

**Table 3:** The correlation between FLIR and collapse.

	Hips	FLIR (%)	z	*p*
Progressive collapse				
Progressive collapse group	25	10.57 (6.51,12.04)	-6.502	0.000
Non-progressive collapse group	56	30.72 (24.85,41.51)		
Terminal collapse				
Terminal collapse group	34	7.80 (2.40,13.63)	-5.617	0.000
Necrotic repair group	35	21.32 (17.25,28.04)		

**Table 4:** Analysis of Independent Risk Factors for APIR and FLIR.

	Univariate P Value	OR (95% CI)	Multivariate P Value	OR (95% CI)
Progressive collapse				
Age (yr)	0.922		/	
18-35				
36-55				
>55				
Sex	0.616		/	
Females				
Males				
Associated factor	0.338		/	
Corticosteroid				
Alcohol				
Idiopathic				
APIR	0.000	0.869 (0.812-0.930)	0.018	0.886 (0.801-0.980)
FLIR	0.000	0.780 (0.699-0.870)	0.000	0.783 (0.690-0.888)
Terminal collapse				
Age (yr)	0.789		/	
18-35				
36-55				
>55				
Sex	0.676		/	
Females				
Males				
Associated factor	0.117		/	
Corticosteroid				
Alcohol				
Idiopathic				
APIR	0.000	0.796 (0.709-0.892)	0.001	0.823 (0.733-0.924)
FLIR	0.001	0.910 (0.859-0.964)	0.025	0.941 (0.893-0.992)

**Figure 3:**
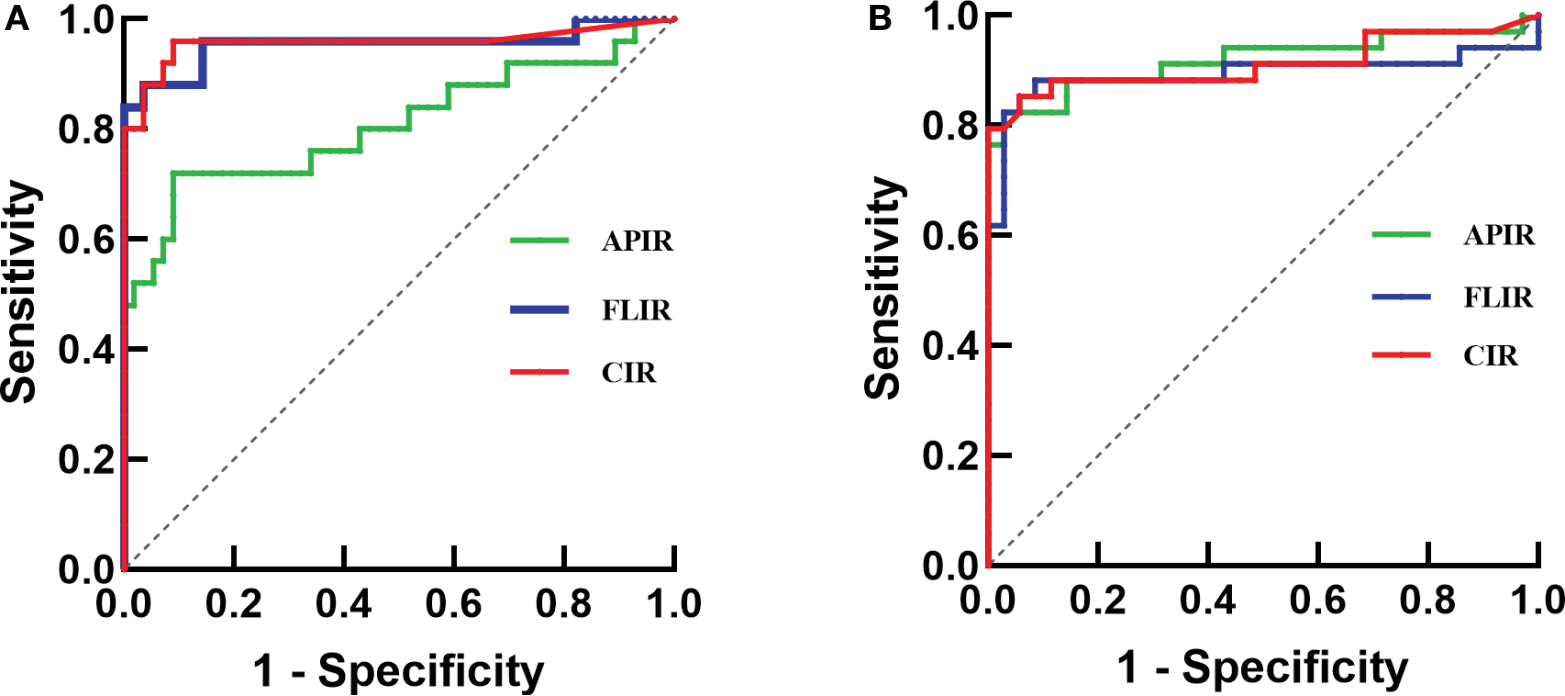
ROC curve analysis of APIR, FLIR and CIR. **(A)** Progressive collapse as an endpoint. **(B)** Terminal collapse as an endpoint.

The combined intact ratio (CIR) was an ROC model for the joint diagnosis of APIR and FLIR. For the progressive collapse group, ROC curve analysis showed the cutoff value of 19.83% (AUC=80.8%, sensitivity=72%, specificity=91%, Youden index=0.63) for APIR, the cutoff value of 17.63% for FLIR (AUC=95.4%, sensitivity=88%, specificity= 96%, Youden index=0.84), CIR (AUC=96.4%, sensitivity=96%, specificity=91%, Youden index=0.87), had a higher diagnostic value for collapse (**Table 5**, **Figure 3A**). For the terminal collapse group, the cutoff values of APIR and FLIR were 16.60% and 16.09%, respectively. Compared with the two, the CIR had a more balanced diagnostic value for terminal collapse (**Table 5**, **Figure 3B**).

**Table 5:** The ROC Curve Analysis of APIR, FLIR and CIR.

	Cutoff	AUC (%)	Sensitivity/Specificity	Youden’s index
Progressive collapse				
APIR	19.83%	80.8	72%/91%	0.63
FLIR	17.63%	95.4	88%/96%	0.84
CIR	0.31	96.4	96%/91%	0.87
Terminal collapse				
APIR	16.60%	91.8	82%/97%	0.79
FLIR	16.09%	89.3	88%/91%	0.80
CIR	0.58	91.2	85%/94%	0.80

CIR, Combined Intact Ratio is the joint ROC of APIR and FLIR; AUC, Area Under Curve.

**Figure 4:**
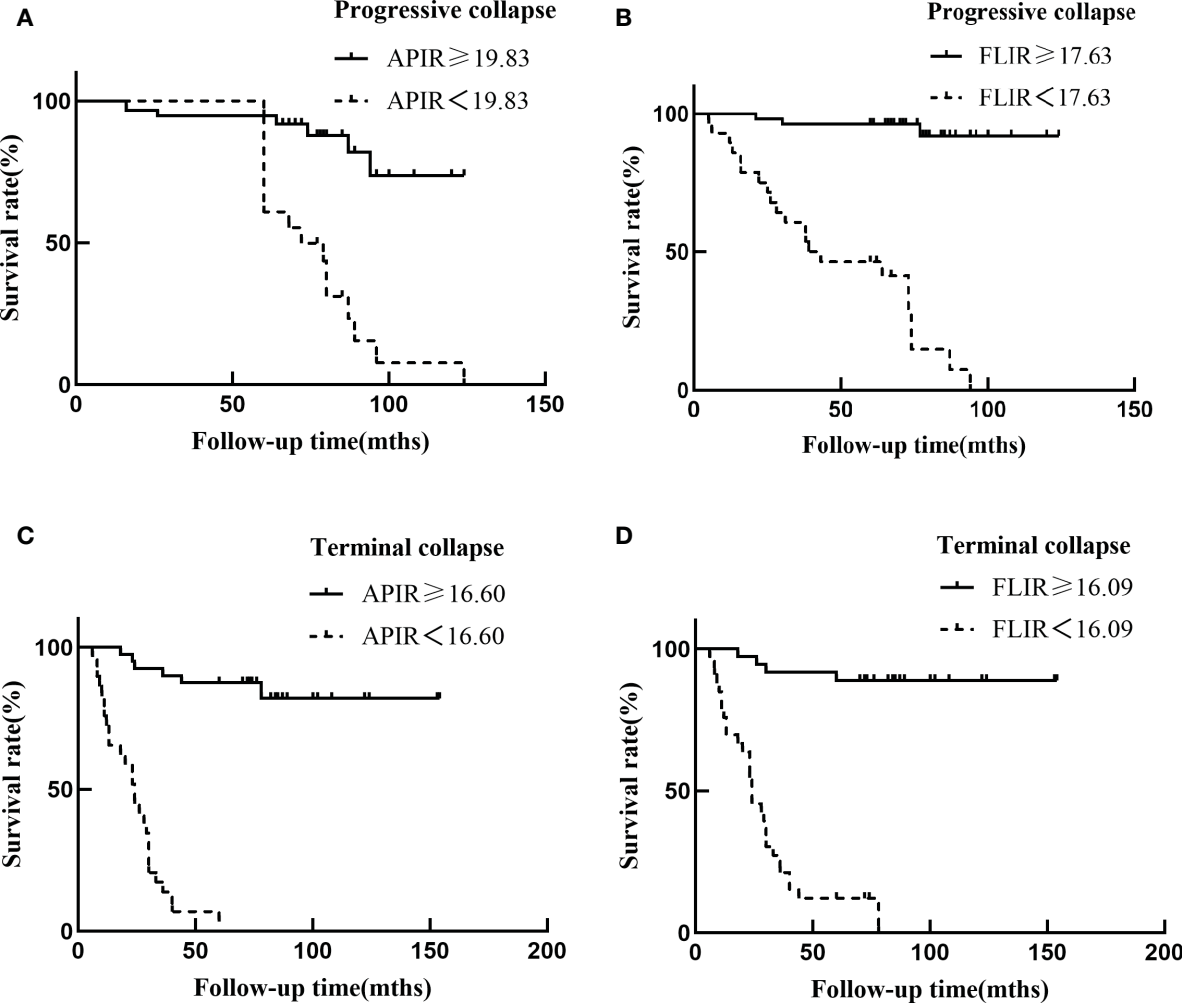
Kaplan Meier femoral head survival curves **(A-D)**. Smaller values of APIR and FLIR (dashed lines) indicate that the necrotic lesion boundary is closer to the outer edge of the acetabulum, which means less intact femoral head is available to bear weight leading to reduced survival of the femoral head.

The K-M survival analysis was performed with the cutoffs value of APIR and FLIR as noted. For progressive collapse, the overall survival rates were 94.8% at 6 years, 73.8% at 10 years when APIR≥19.83% (**Figure 4A**), 96.2% at 6 years, and 91.9% at 10 years when FLIR≥17.63% (**Figure 4B**). For terminal collapse, when the 6-year and 10-year survival rates were 87.5% and 82.0%, respectively, when APIR≥16.60% (**Figure 4C**), and were both 88.9% when FLIR≥16.09% (**Figure 4D**).

In summary, the necrotic lesion boundary classifications predict relatively good prognosis for of type Aa, type Ab, type Ae, type Ba, type Bb, type Be, type Ea, type Eb and type Ee and non-surgical approaches are recommended. On the contrary, type Da, type Db, type Dc, type Dd, type De, type Ad, type Bd, type Cd and type Ed are predicted to have poor prognosis, which would require hip preservation surgery or THA. Lastly the prognosis for type Ac, type Bc, type Ca, type Cb, type Cc, type Ce, and type Ec are undetermined and need to be further accessed based on our model (**Table 6**).

**Table 6:** The prognosis prediction and treatment for the necrosis lesion boundary classification.

The necrosis lesion boundary classification	Type A	Type B	Type C	Type D	Type E
Type a	Good prognosis/non -surgical treatment	Good prognosis/non-surgical treatment	APIR≥19.83%: good prognosis/non-surgical treatment; APIR<19.83%: poor prognosis/hip preservation surgery or THA	Poor prognosis/hip preservation surgery or THA	Good prognosis/non-surgical treatment
Type b	Good prognosis/non -surgical treatment	Good prognosis/non-surgical treatment	APIR≥19.83%: good prognosis/non-surgical treatment; APIR<19.83%: poor prognosis/hip preservation surgery or THA	Poor prognosis/hip preservation surgery or THA	Good prognosis/non-surgical treatment
Type c	FLIR≥17.63%: good prognosis/non -surgical treatment; FLIR<17.63%: poor prognosis/hip preservation surgery or THA	FLIR≥17.63%: good prognosis/non-surgical treatment; FLIR<17.63%: poor prognosis/hip preservation surgery or THA	APIR≥19.83% and FLIR≥17.63%: good prognosis/non-surgical treatment; APIR<19.83% and/or FLIR<17.63%: poor prognosis/hip preservation surgery or THA	Poor prognosis/hip preservation surgery or THA	FLIR≥17.63%: good prognosis/non-surgical treatment; FLIR<17.63%: poor prognosis/hip preservation surgery or THA
Type d	Poor prognosis/hip preservation surgery or THA	Poor prognosis/hip preservation surgery or THA	Poor prognosis/hip preservation surgery or THA	Poor prognosis/hip preservation surgery or THA	Poor prognosis/hip preservation surgery or THA
Type e	Good prognosis/non -surgical treatment	Good prognosis/non-surgical treatment	APIR≥19.83%: good prognosis/non-surgical treatment; APIR<19.83%: poor prognosis/hip preservation surgery or THA	Poor prognosis/hip preservation surgery or THA	Good prognosis/non-surgical treatment

## Discussion

ONFH is a disabling disease that largely affects middle-aged and young patients. If not intervened in time, some patients with ONFH can develop femoral head collapse or hip osteoarthritis, both of which indicate for THA (25, 26). For younger patients, serious complications of THA, and poor durability of hip prosthesis make hip preservation treatment especially beneficial (27). The prevailing belief is that the occurrence of collapse predicts a poor prognosis in ONFH, and hip preservation surgery or THA are the only treatment options (13–21, 28). This view has led to an emergence of studies on assessing risk for collapse in early stage of ONFH, in the hopes for early intervention strategies (3–10, 12, 29–31). The aim of these studies was to select an appropriate hip preservation strategy based on the probability of hip collapse. However, we observed a wide range of variability in the progress of ONFH patients with collapsed femoral heads clinically. We hypothesized that the necrotic lesion boundary better predicts the prognosis.

Consistent with clinical observations, we found that out of the 106 hips in ARCO stage II, 31 hips collapsed with further progression. 75 hips had no collapse or collapse with repair of the necrotic areas. Out of the 97 hips in ARCO stage IIIA, the collapse continued to progress in 58 hips while the necrotic areas were repaired in 39 hips (**Table 1**). We then classified the necrotic lesion boundary by APIR and FLIR, a quantification of the lesion boundary. These results suggested that the necrotic lesion boundary classification turned out to be a key factor determining the outcome in both the progressive collapse and terminal collapse groups, suggesting the predictive value of APIR and FLIR for ONFH prognosis (**Tables 1**-**5**, **Figures 3**, **4**). These results prompted that the prognosis and treatment of type Ac, type Bc, type Ca, type Cb, type Cc, type Ce, and type Ec need to be further accessed according to the cutoff values of APIR and FLIR, and the rest of the types had relatively clear prognosis and treatment options. Taken together, our study creatively proposed a necrosis lesion boundary classification, which predicts prognosis and inform treatment options (**Table 6**).

Literature research have further revealed prior studies that found associations between the anterior necrotic lesions and femoral head collapse (8, 9, 32–34). These studies suggest that not only the lateral necrotic lesions but also the anterior necrotic lesions contribute to the prognosis potential of ONFH and should be taken into consideration when making clinical decisions.

In regards to evaluating the necrotic lesions, even though Computed tomography (CT) and Magnetic Resonance Imaging (MRI) can provide higher-resolution images, x-rays are currently still the most frequently used clinical examination imaging modality in China due to their low cost beneficial for the large population base. X-rays also have the ability to clearly show the anatomical relationships between bones, making them the best tools for identifying the boundary of necrotic lesions. Several studies have confirmed the value of an FL view on top of a traditional AP view of the X-rays for a clear evaluation of the anterior necrotic lesions (3, 12, 35). Therefore, both AP and FL views of the lesions need to be taken into account when prognosis is determined. The necrosis lesion boundary classification by X-rays described in this work has taken into account both the AP and FL views, which improved the evaluation accuracy in our analyses.

Current commonly used staging such as Ficat staging, Steinberg staging and ARCO staging only reflect the progression of ONFH (whether there is collapse or arthritis) compared to the necrosis lesion boundary classification, while ignoring the location of the necrotic lesion which is considered to be predictive of prognosis. In addition, although the Japanese Investigation Committee (JIC) classification is widely accepted across the world, it is limited in its ability to predict the prognosis of ONFH since it is based solely on the AP view and ignores the three-dimensional anatomy of the femoral head. In general, compared to other classifications, the necrosis lesion boundary classification is a more appropriate new classification to determine the prognosis of ONFH.

This study has several limitations. First, the necrosis lesion boundary classification cannot be evaluated for ARCO stage I. Second, this classification does not take into account the size of the necrotic areas, which could potentially affect prognosis. Finally, the sample size of this study is relatively small, and real-life situations may have more complicated considerations. However, these limitations do not obscure the strengths of this study. The patients with non-progressive collapse group and necrotic repair group included in this study were followed up for at least 5 years, maximizing the accuracy of outcome determination. In addition, the necrotic lesion boundary classification is a novel classification that quantify lesion boundary based on both AP and FL views of x-rays

## Conclusion

Taken together, the occurrence of collapse is an oversimplified predictor for ONFH prognosis. The present study demonstrates that the necrosis lesion boundary has a high value to predict ONFH prognosis and to inform clinical treatment options.

## Data availability statement

The raw data supporting the conclusions of this article will be made available by the authors, without undue reservation.

## Ethics statement

This study was approved by the institutional review board of the First Affiliated Hospital of Guangzhou University of Chinese Medicine (No. JY [2021]165). The patients/participants provided their written informed consent to participate in this study.

## Author contributions

Conceptualization: YF and ZC. Data extraction and quality assessment: YZ and XL. Software: YF and YL. Formal analysis: JZ and HF. Validation: CZ, WH and ZC. Writing: YF and CZ. All authors contributed to the article and approved the submitted version.
